# Studies of structural determinants of substrate binding in the Creatine Transporter (CreaT, SLC6A8) using molecular models

**DOI:** 10.1038/s41598-020-63189-z

**Published:** 2020-04-10

**Authors:** Claire Colas, Giulia Banci, Riccardo Martini, Gerhard F. Ecker

**Affiliations:** University of Vienna, Department of Pharmaceutical Chemistry, Vienna, Austria

**Keywords:** Drug discovery, Computational biology and bioinformatics

## Abstract

Creatine is a crucial metabolite that plays a fundamental role in ATP homeostasis in tissues with high-energy demands. The creatine transporter (CreaT, SLC6A8) belongs to the solute carrier 6 (SLC6) transporters family, and more particularly to the GABA transporters (GATs) subfamily. Understanding the molecular determinants of specificity within the SLC6 transporters in general, and the GATs in particular is very challenging due to the high similarity of these proteins. In the study presented here, our efforts focused on finding key structural features involved in binding selectivity for CreaT using structure-based computational methods. Due to the lack of three-dimensional structures of SLC6A8, our approach was based on the realization of two reliable homology models of CreaT using the structures of two templates, i.e. the human serotonin transporter (hSERT) and the prokaryotic leucine transporter (LeuT). Our models reveal that an optimal complementarity between the shape of the binding site and the size of the ligands is necessary for transport. These findings provide a framework for a deeper understanding of substrate selectivity of the SLC6 family and other LeuT fold transporters.

## Introduction

Creatine plays an essential role for ATP homoeostasis in tissues with high-energy demand such as brain, heart and skeletal muscle. Creatine is intracellularly converted into phosphocreatine by the creatine kinase, which then serves as ATP storage. The creatine transporter CreaT is responsible for the uptake of this important metabolite. Mutations or malfunction of CreaT lead to creatine deficiency in the brain, which ultimately can lead to severe neurological diseases such as mental retardation and epilepsy^1,2^. CreaT is a member of the solute carrier transporter 6 family (SLC6), and specifically the GABA transporters (GATs) subgroup, which in addition includes the taurine transporter TauT, the betaine transporter BGT1, and the GABA transporters GAT1, GAT2, and GAT3. The SLC6 members are symporters, since they couple transport of Na^+^ in the same direction as the substrate and are classified as secondary active transporters, as they use the electrochemical potential difference across the cell membrane of Na^+^ as energy source to transport their substrates.

**Figure 1:**
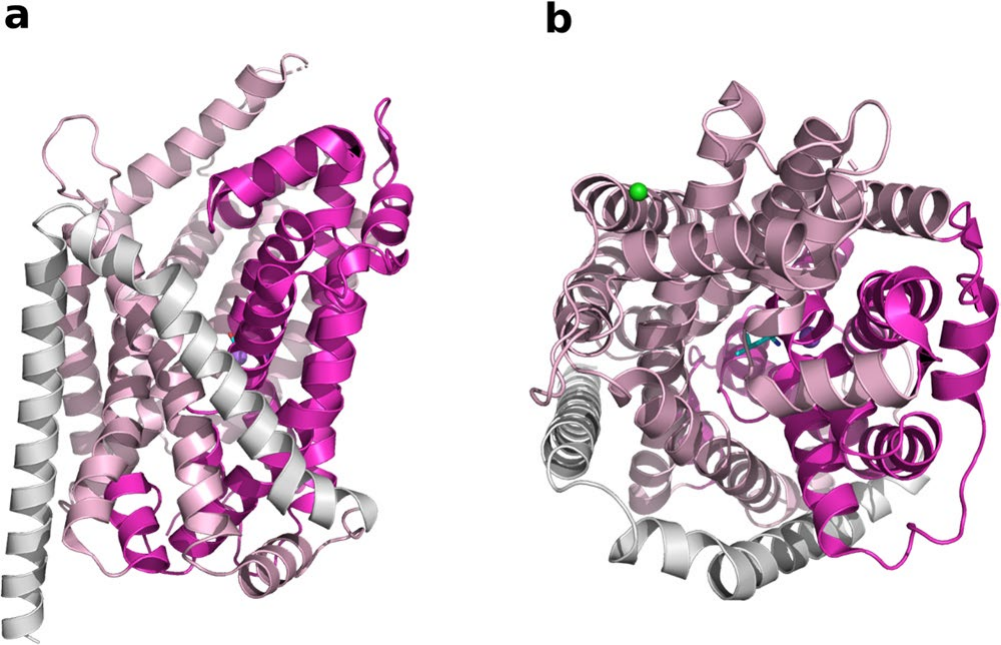
Three-dimensional structure of LeuT. The scaffold and transport domain are colored in light and dark pink, respectively. The picture was generated with Pymol^52^. LeuT (PDB ID 2A65) is shown from the side (**a**) and top view (**b**). The bound leucine is shown in sticks and the sodium and chloride ions in purple and green spheres, respectively.

The SLC6 transporters mediate the intracellular uptake of neurotransmitters, metabolites and amino acids. Mutations or malfunction of these transporters are associated with various diseases. Particularly the monoamines subgroup (that include the serotonin, dopamine and norepinephrine transporters) have been associated to neurological disorders such as anxiety or depression^3,4^. The GABA transporters are also important targets for the treatment of epilepsy or stroke^5,6^. Therefore, these proteins have been extensively studied due to their pharmacological impact. The transport operated by the SLC6 family is defined as an alternating gated pore mechanism, common to all transporters sharing the LeuT fold^7–9^. In fact, the prokaryotic transporter LeuT has been largely studied as a representative transporter of this family, which fundamentally helped to elucidate the molecular mechanisms of transport^10–12^. Additionally, several structures of the human serotonin transporter (hSERT)^13–15^, as well as the *Drosophila* DAT (dDAT)^16^ have been solved, providing precious insight into the fold and mechanism of transport of this family of proteins. The LeuT fold consists of 12 transmembrane (TM) helices, with 10 of these helices constituting the core of the transporter that are connected by loops and arranged in two 5-TM pseudo symmetric inverted repeats. In the transport process, the transporter alternates between outward open and inward open conformations. Particularly, a dynamic bundle domain (constituted of TM1,2 and TM6,7) alternates conformations to allow the entering and release of the substrate, against the more rigid scaffold domain (constituted of TM3-5 and TM8-10) (Fig. 1).

As mentioned previously, the pharmacology of the SLC6 transporters has been studied for many years, however, a lot remains to be understood. Specifically, the molecular mechanisms defining the substrate specificities within the GATs subfamily is unclear, but is essential to decipher in order to increase the success rate in drug discovery. This is particularly challenging, since they share high sequence identities with each other, ranging from 50 to 90% in their binding sites. Thus, efforts have been made to structurally characterize the GABA transporters^17–19^.

In this study, we combined computational analysis with published experimental data to increase our understanding of the creatine transporter specificity and selectivity, particularly among guanidine like ligands. We first present the homology model of the creatine transporter in two distinct conformations of the transport cycle, i.e. in outward occluded and outward open conformations. These models have been built using the three-dimensional structures of LeuT and hSERT as templates, respectively. We then performed induced fit docking of known CreaT ligands. These results permitted to highlight the optimal complementarity of the size and shape of the binding site with the size of the ligands. Finally, we discuss how our findings provide a new perspective into the SLC6 transporter family, giving insight into the importance of the shape, volume and physico-chemical properties of the binding site and how it directly influences substrate specificity. In particular, the presence of π-helices in SLC6 transporters is addressed and studied.

## Results

### CreaT homology models

**Figure 2:**
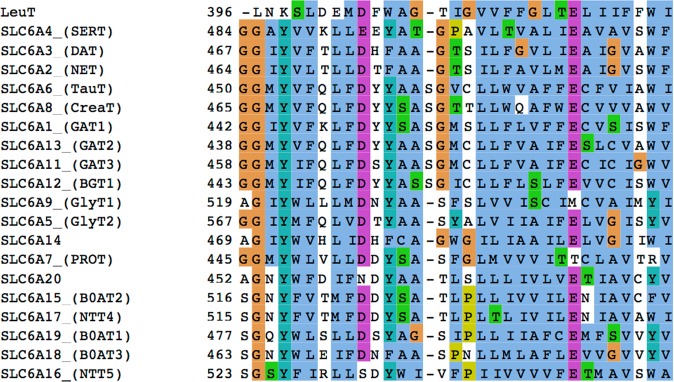
Multiple sequence alignment of the SLC6 family. The alignment of the TM10 is shown, to highlight the insertion present in the GAT subgroup. The representation was made with Jalview^53^.

Having access to various conformational states of the transport cycle is an essential step of productive structure-based studies on Solute Carriers. We created two distinct models of the creatine transporter in outward open and outward occluded conformations of the transport cycle by using hSERT and LeuT as templates (Methods). These two templates were selected because of a similar predicted fold, the high sequence identity of hSERT with CreaT (44%) and the outward occluded conformation of LeuT, more suitable to accommodate substrates. However, the presence of an additional amino acid – S479 - in TM10 of CreaT in the multiple sequence alignment (Methods, Fig. 2) requires particular attention. All GATs including CreaT and TauT present this additional amino acid in TM10, next to the orthosteric binding site of the transporters. This insertion has been reported and discussed for the GABA transporters^17,18,20^ and was in fact described as a π-helix in GAT1^17^.

However, to the best of our knowledge this issue was never addressed for CreaT. As this TM10 insertion is located in the binding site of the transporter, building a reliable model requires particular attention. Due to lack of structural data, it is still an open discussion whether this insertion generates a more unwound region (loop) or a π-helix. In proteins, distinct types of helices can be found, such as alpha helices, 3.10 helices and π-helices. Helices are characterized by number of residues (i) per turn and the hydrogen bonds connectivity between the carbonyl moiety of one residue and the amino moiety of another. An alpha helix contains 3.6 residues per turn and the hydrogen bonding connectivity is i → i + 4. A 3.10 helix is tighter with a hydrogen bonding connectivity of i → i + 3. Conversely, a π-helix contains an average of 7 amino acids per turn and is looser, with a hydrogen bonding connectivity of i → i + 5. Generally, the presence of a π-helix is considered structurally unfavorable because (i) the dihedral angles are unfavorable, (ii) the hole at the center of the helix is 1 Å wide which results in a loss of VdW interactions, and (iii) four residues need to be correctly aligned to allow the i + 5 hydrogen bonding.

We hypothesized, however, that the insertion observed in TM10 creates a π-helix in CreaT for the following reasons. First, TM10 belongs to the scaffold domain of the LeuT fold and thus, is unlikely to contain an unwound region. In fact, only two broken helices have been characterized in transporters presenting a LeuT fold, i.e. TM1 and TM6, which are both in the transport domain. Their unwound regions have been shown to allow the conformational change of the transporters from outward to inward facing states, necessary to facilitate the binding and release the substrate^21^. Second, the presence of a π-helix creates a narrower pathway into the binding site, directly impacting substrate specificity. Furthermore, previous work on GAT1 suggested that the π-helix resulting from this insertion induced an optimal packing of the binding site for efficient substrate:ion coupling^17^.

**Figure 3:**
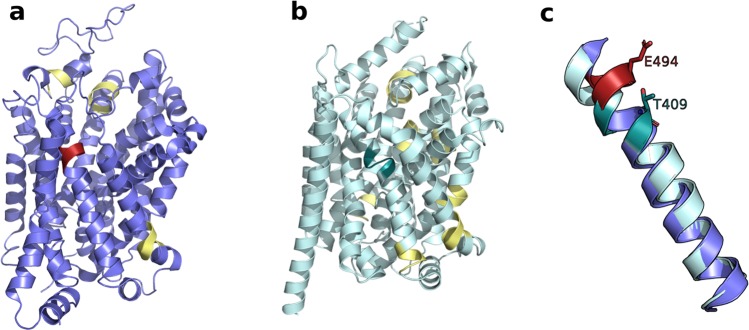
Detected π-helices in hSERT and LeuT. The DSSP tool (Methods) was used to detect π-helices in hSERT (**a**) and LeuT (**b**), shown in blue and cyan cartoons, respectively. The identified π-helices are colored in yellow, with the exception of the one in TM10, colored in red in hSERT and dark cyan in LeuT. The superposition of the TM10 regions used for modeling reveal a shift of the π-helix found in TM10 between the two transporters (**c**).

To investigate this issue further, we used the DSSP tool (Methods) to detect the presence of π-helices in SERT and LeuT (Fig. 3(a,b)). Four π-helices were detected in hSERT and eight in LeuT, with one in TM10 for each transporter. Interestingly, the position of the π-helix in the TM10 of hSERT is shifted by one helix turn when compared to the one of LeuT (Fig. 3(c)) and the GATs subfamily. In fact, in hSERT, the additional amino acid creating the π-helix is E494, whereas it is T409 in LeuT. Surprisingly, the π-helix detected in TM10 of LeuT is located near the insertion observed in the GAT subgroup (Fig. 2), but is not marked as an insertion in the multiple sequence alignment. When building homology models, it is known that the sequence alignment needs to be carefully inspected and manually refined for correct alignment of motifs and gaps.

**Figure 4:**
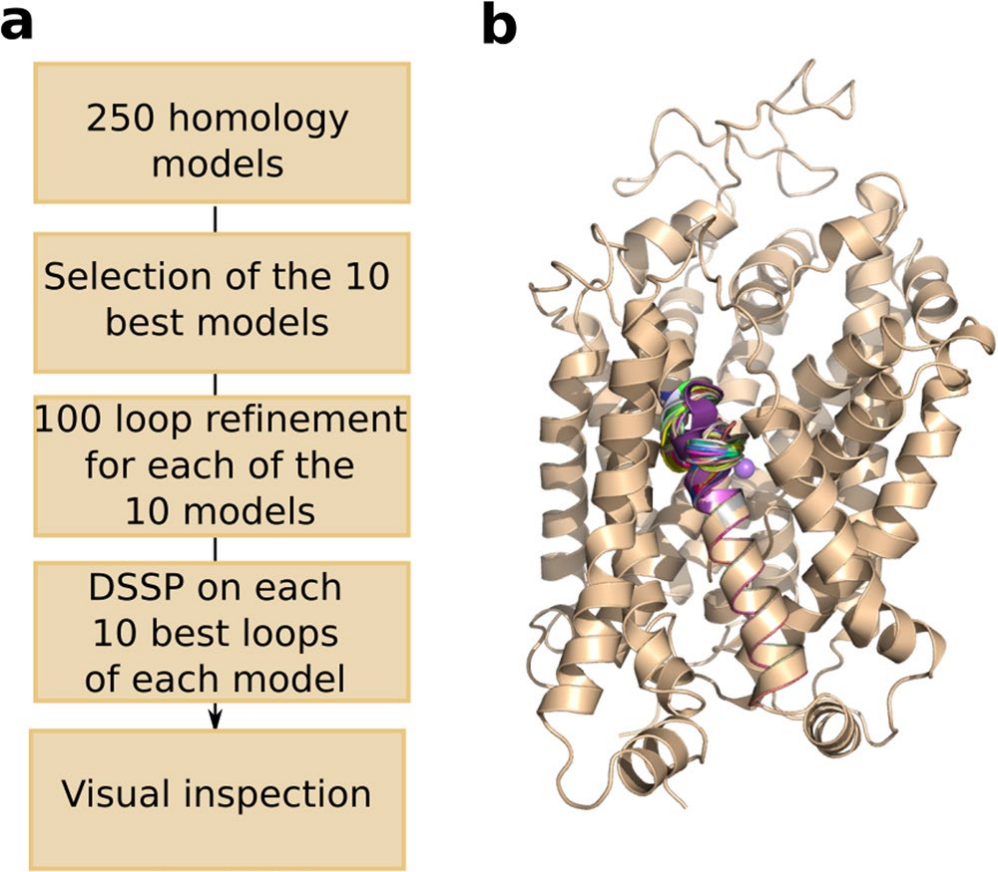
Homology modeling protocol. We modeled CreaT in the outward open and outward occluded conformation using the same procedure (**a**) described in details in the Methods section. Particular attention was carried in the loop refinement step. Panel (b) shows the 100 loops generated for the best model in the outward open conformation. The majority of CreaT is represented in beige cartoons, with the exception of the 100 loops in various cartoon colors.

**Figure 5:**
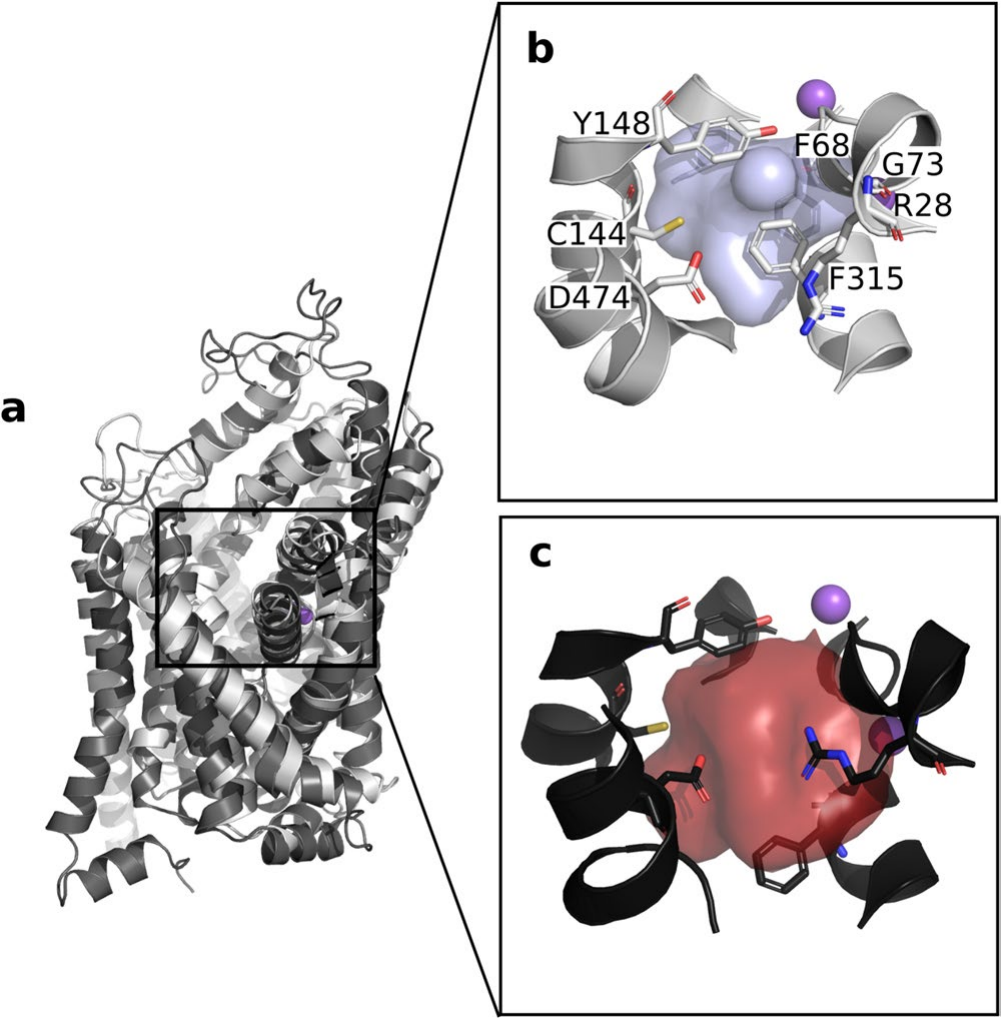
CreaT models in two distinct conformations. (**a**) The two models are superposed, with the outward open and outward occluded conformations shown in dark and light gray cartoons, respectively and sodium ions in purple spheres. The binding pockets are shown in blue in the outward occluded binding site (**b**) and in red in the outward open site (**c**), with the residues enclosing the pockets represented in sticks. The sodium ions are shown in purple spheres. The binding pockets have been calculated with POVME 2.0^54^, using an inclusion radius of 6 Å around the center of the binding site.

In this particular case, 250 models were generated for each conformations of CreaT using Modeller^22^, and evaluated by the statistical potential Z-DOPE score implemented in Modeller (Methods)^23^. A loop refinement protocol was applied to the 10 best models around the additional S479 residue (Fig. 4, Methods). We compared the models of CreaT in two distinct conformations of the transport cycle, i.e. outward open and outward occluded conformations. Both models present a LeuT fold, as expected from their respective templates, i.e. twelve transmembrane helices arranged in a scaffold and bundle domain (Fig. 5a). Key functional residues are located at conserved positions in the binding site accordingly to what has been described in the literature. For example, Y148 and F315 constitute the hydrophobic lid enclosing the binding site, while D474 and R28 form the extracellular gate (Fig. 5(a,b)). As observed from comparing the templates, the main difference between the two conformations is a tilting of two broken helices TM1 and 6 on the extracellular side, as well as a tilt of the conserved Y148, acting as the hydrophobic extracellular lid. These changes result in a significant decrease of the binding site volume, i.e. 117 Å^3^ in the outward occluded conformation as opposed to 349 Å^3^ in the outward open conformation (Fig. 5).

Finally, particular attention was paid on C144. In fact, this residue located in TM3 is not conserved within the SLC6 family, and unique to CreaT. In all other GATs subfamily members, this position is substituted by a Leucine, and by a Valine in the monoamine transporters. Thus, C144 is suspected to be involved in the binding specificity of CreaT. In fact, mutagenesis studies have been conducted in a previous study, showing that mutating C144 to Leucine, in combination with two other substitutions in CreaT (i.e. F68Y and A318G) led to a gain of GABA transport^24^. Furthermore, a series of mutations of C144 to Serine, Alanine and Leucine provided essential information on the importance of this residue^25^. First, this study permitted to emphasize the importance of the size and physico-chemical properties of the residue at this position. In particular, affinity of CreaT substrates decreased in mutants with long and hydrophobic side chains at this position. Second, the inactivation of CreaT by low concentrations of 2-aminoethyl methanethiosulfonate (MTSEA) suggested that C144 is likely to be deprotonated and solvent exposed to quickly create a covalent disulfide bond with the sulfur of the reagent^25,26^. Thus, C144 was deprotonated in our models to conduct our dockings (Methods).

### Rationalizing substrate specificity

Structure based analysis studies are powerful to functionally characterize protein-ligand interactions and identify essential binding residues. In particular, the description of these structural determinants such as the polarity, protonation and shape of the binding site can influence greatly the differential binding and transport activities of small molecules. Here, we combine several computational tools complemented by available experimental data to guide the characterization of the structural key determinants of binding in the creatine transporter.

CreaT substrates present a carboxylate and guanidine group linked by a 2–3 carbons chain^27,28^. Strikingly, despite a similar scaffold, CreaT ligands present a wide range of activities (Table 1). Thus, we investigated the influence of the carbon chain length on the transport activity. We performed induced fit docking of five known CreaT ligands that present distinct carbon chain length and transport activities (Table 1) in the binding site of our final outward occluded model of CreaT, more appropriate to depict the ligands interactions with the binding site (Methods). This type of docking allows the flexibility of the residues side chains and thus, the accommodation of the binding site to the ligands.

**Table 1:** Docked CreaT ligands. ^*a*^Refers to the name of the ligand. ^*b*^2D sketch representing the two-dimensional structure of the compound. ^*c*^Number of carbons constituting the linker between the carboxylate and guanidine moieties. ^*d*^Length in Å of the carbon chain linking the carboxylate to the guanidine groups. ^*e*^IC_50_ refers to the experimental IC_50_ of each compound, with the exception of the natural substrate creatine, where Km is reported. These values are reported as found in literature^55,56^, with the GAA IC_50_ value reported for the rabbit CreaT^57^. ^*f*^Indicates the Glide docking score calculated from the Induced fit dockings. ^*g*^Reports the MMGBSA values calculated with the Schrödinger package.

CreaT ligands^*a*^	2D Sketch^*b*^	Number of carbon atoms of the linker^*c*^	Length (Å)^*d*^	IC_50_^*e*^	Glide score from IFD^f^	MMGBSA^*g*^ (kcal/mol)
Creatine		1	3.8	Km = 0.2 mM	−5.43	−24.68
ATPCA		2	4.3	66 µM	−7.411	−47.02
Beta-Guanidinopropionate (Beta-GPA)		2	5	44 µM	−6.86	−43.68
Gamma-Guanidinobutyric acid (Gamma-GBA)		3	5.8	697.9 µM	−6.58	−45.57
Guanidinoacetate (GAA)		1	3.7	712 µM	−5.24	−25.79

**Figure 6:**
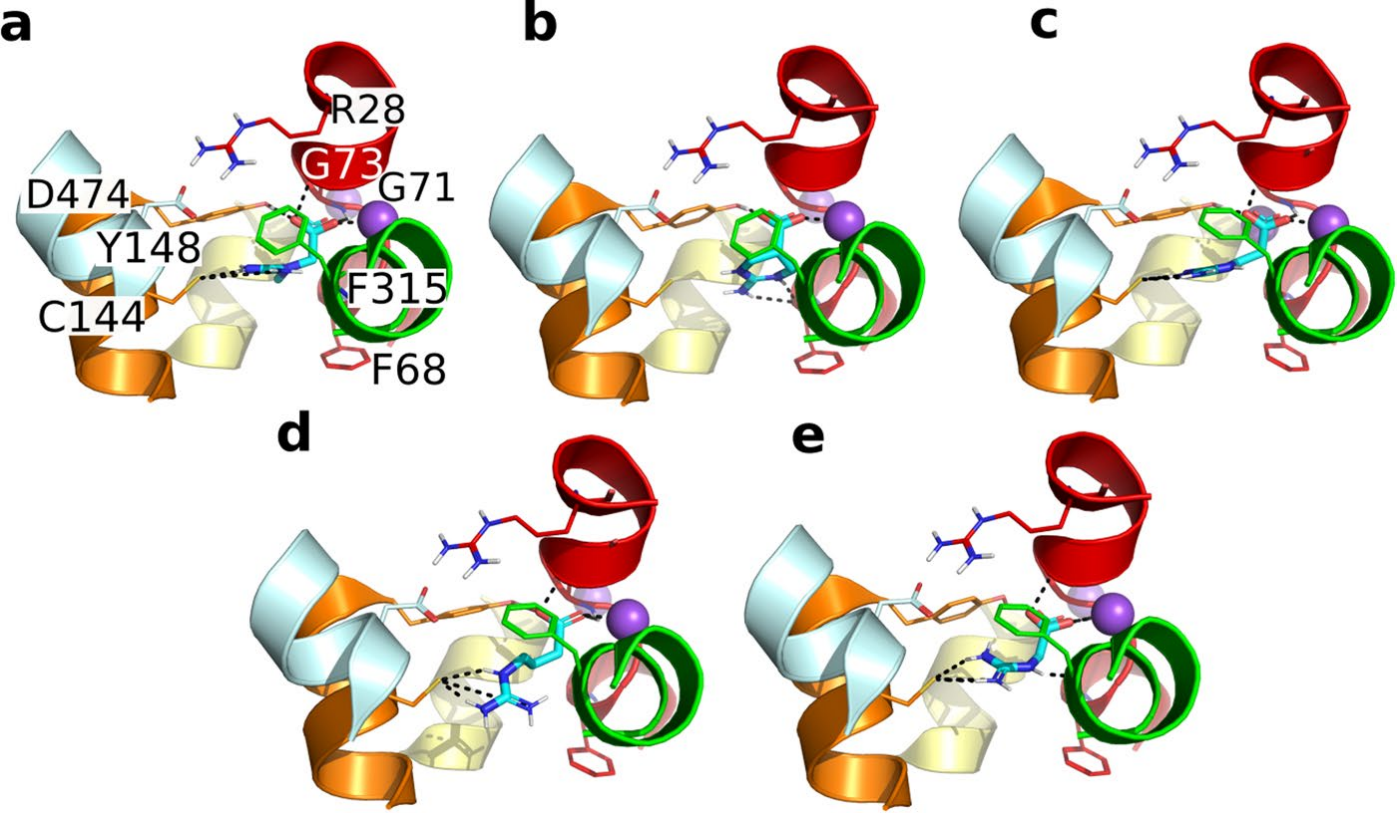
Binding poses of five known CreaT ligands. Best docking pose resulting from the induced fit docking of each ligand, i.e. creatine (**a**), ATPCA (**b**), Beta-GPA (**c**), Gamma-GBA (**d**) and GAA (**e**). The ligands are shown in cyan sticks. CreaT is shown in cartoons, with TM1, 3, 6, 8 and 10 colored in red, orange, green yellow and cyan respectively. Residues interacting with the ligands are shown in sticks and the hydrogen bonds, salt bridges and ionic interactions in black dashed lines. The sodium ions are shown in purple spheres.

For each ligand, the docking poses presenting the best Emodel scores were selected to be compared to the poses of the other ligands. The interactions observed are consistent throughout the complexes. Notably, the carboxyl moiety establishes polar interactions with the backbone of G71, G73, the hydroxy group of Y148 and coordinates the Na^+^ (Fig. 6). This tyrosine is conserved among the GABA subfamily as well as in hSERT and LeuT. In fact, previous docking studies on a homology model of hBGT1 showed polar interactions of the carboxylic group of GABA with the corresponding residue Y133, as well as G57 and L56^18^. The guanidine group establishes a salt bridge with the deprotonated C144 and π-π interactions with either Y148 or F315 (Fig. 6).

To rationalize the activities of the ligands, we evaluated the MMGBSA binding energies from the Schrödinger package, known to be more accurate than docking scores^29^. In fact, this method has been proven in previous studies to be appropriate to analyze the interactions of ligands with peptide transporters^30–32^. It should be noted, that the generated predicted binding affinity values are used to be qualitatively compared to the experimental IC_50_, and are not expected to reproduce the absolute values, as was previously shown in previous studies^30–32^.

Interestingly, ATPCA and Beta-GPA - the two best inhibitors - and Gamma-GBA have similar MMGBSA values. This was unexpected, since Gamma-GBA is a weaker inhibitor than ATPCA and Beta-GPA. These observations suggest that an optimal length of a two carbons linker between the carboxy and guanidine groups is required. Gamma-GBA has a three-carbon linker, which suggests weaker interactions in the binding site, despite the predicted MMGBSA value.

Finally, GAA has only a one-carbon chain length. GAA has a similar predicted binding energy as creatine, which corresponds to the observed IC_50_. We assume that GAA can only establish weak interactions with the binding site residues because of its small size.

## Discussion

In human and mammals, creatine is an important metabolite used as energy storage for skeletal muscles and heart. Creatine is synthesized in kidneys and liver, brought to the target tissues through the blood stream and transported intracellularly by the creatine transporter CreaT. The goal of this project was to structurally characterize CreaT by building reliable homology models and rationalize the transport activities of known ligands using computational tools, complemented by available experimental data found in literature. Three key findings emerge from this study.

First, our homology models provide insight into the structural determinants characterizing the substrate selectivity of CreaT. Two features in particular seem to be essential. First, the presence of a π-helix in TM10 provides a specific packing of the binding site. The fact that the π-helix is not placed at a similar position in hSERT suggests that this feature probably influences substrate selectivity between the distinct subfamilies of SLC6. The second key feature is the deprotonated cysteine 144, a residue specific to CreaT located on TM3. Interestingly, it has been suggested that TM3 might be involved in substrate recognition, notably due to a very conserved Tyrosine (Y148 in CreaT, Y176 in hSERT)^33,34^. The presence of this cysteine unique to TM3, and its essential role in CreaT ligand binding reinforces the hypothesis that this area of the binding site is important for the transport function.

Second, our dockings rationalized the substrate properties of known CreaT ligands. It was known that optimal substrate properties were observed for compounds with a carboxylate and a guanidine group separated by 2–3 carbons^28^. In this study, our results describe the structural basis for the distinct activities observed for five known CreaT ligands (Table 1). Our docking reveal that an optimal length of carbon linker of approximately 4.5–5 Å is required for the guanidine moiety to establish a hydrogen bond with C144 and the carboxylate moiety with G73 and the Na^+^. Two possible reasons for this limitation come to mind: (i) our homology models in two conformations of the transport cycle revealed the flexibility of the binding site (Fig. 5). Particularly, the outward occluded binding site is significantly smaller, creating steric hindrance for bulkier ligands. Interestingly, similar tendencies have been revealed in other transporters presenting a distinct fold. For example, in a previous study we showed that ligand specificity of the SLC13 family is influenced by the length of the transported dicarboxylates^35^.

(ii) Our binding poses suggest interactions from the guanidine and carboxylate moieties respectively with the deprotonated C144 and the Na^+^. This suggests the presence of a dipole moment in the binding site between C144 and Na^+^, facilitating the orientation and accommodation of the CreaT ligands. Such binding features have been observed previously in the peptide transporter hPepT1 and its prokaryotic homolog PepT_So_, where very conserved lysine and glutamate residues in their binding sites (i.e. K140 and E595 in hPepT1) create a dipole moment enabling the interactions of the amine and carboxylate moieties of the transported peptides^30,36^. It was hypothesized that such dipole moment is essential for the transport to occur in hPepT1, and the disruption or lack of such interactions was a key structural determinant distinguishing between substrates and inhibitors. It is possible that similar rules monitor the binding properties of the distinct CreaT ligands. In particular, the length of the carbon linker would affect the proper conductivity of the dipole. These observations suggest that, despite the structural variability among SLC transporters, many of them exhibit similarities in their function and molecular recognition.

Furthermore, it should be noted that the five ligands were docked in one unique conformational state. It is possible that Gamma-GBA, a slightly bulkier ligand, binds to a slightly more open conformation, to which we do not have access to – the outward open conformation model being conversely too large. The visual inspection of the binding pose of this ligand shows however that Gamma-GBA is slightly constricted in a small binding site, which would explain why the predicted binding energy of Gamma-GBA does not reflect the discrepancy observed in the experimental IC_50_ with the two most potent inhibitors. This provides additional evidence of the need to have access to multiple conformational states of the transport cycle to conduct structure-based studies on Solute Carriers.

Overall, our results provide indication on how the distinct interactions of these ligands with CreaT induce the differential binding activities. Interestingly, the subtle differences of geometry and flexibility of the compounds do not seem to affect their activities. For instance, ATPCA is slightly shorter and less flexible than the other two-carbon linker Beta-GPA, but exhibit a comparable experimental IC_50_.

Additionally, these results provide a framework for the design of pharmacophores that could be used to discover new specific compounds for CreaT that could be used as chemical tools to further characterize this transporter. This would pave the way towards defining new rules governing the transport specificities and selectivity of the distinct members of the SLC6 family for future drug development.

Finally, our new models provide a new building block to describe further the SLC6 family. A systematic application of homology modeling on each member of the SLC6 family progressively reveals key residues involved in ligand binding and specificity. The presence of π-helices at different locations (Fig. 3) or unconserved residues in the binding site (such as C144 in CreaT) show that, within a similar fold, subtle differences between individual SLC6 transporters alter the physico-chemical properties of the binding sites, which are reflected in the ligand specificities of each transporter.

Furthermore, the increasing number of three-dimensional structures available due to the improvement of cryo-EM methodologies^37,38^ enables the access of SLCs structures in additional conformations of the transport cycle and thus, provides new opportunities to further understand the molecular mechanisms of transport in this pharmacologically important family of transporters.

## Methods

### Homology modeling

We built two homology models of CreaT (Uniprot P48029, SLC6A8_HUMAN), using the crystal structure of hSERT in an outward open conformation (PDB ID:5I73, resolution 3.24 Å)^15^ and of LeuT in an outward occluded state (PDB ID 2A65, resolution 1.65 Å)^39^ as templates. CreaT presents sequence identities of 44% and 21% with hSERT and LeuT, respectively.

We first built a multiple sequence alignments using Promals3D^40^ containing all the human SLC6 transporters as well as, LeuT. Additionally, we superposed the three-dimentional structures of hSERT and LeuT for visual inspection. As shown by the sequence alignment, the TM10 presents an extra amino acid in all GATs (S479 in CreaT, Fig. 2), which has been previously hypothesized to constitute a π-helix^17^. We used the program DSSP to detect the presence and position of π helices in hSERT, and LeuT^41^. pi-HUNT^41^ was then used to analyze the DSSP files generated for hSERT and LeuT to detect π-helices. This program predicted the presence of a total of 8 π-helices in LeuT, and 4 π-helices in hSERT (Fig. 3(a,b)), including one π-helix in TM10 in each case (Fig. 3(c)). Thus, we paid particular attention to this region of our model and conducted a loop refinement protocol obtain a better defined π-helix.

First, using MODELLER version 9.22^42^, we built the homology models of the creatine transporter using hSERT and LeuT as templates. 250 models were generated and evaluated by looking at their normalized DOPE score^23^. The 10 best models (with Z-DOPE scores from −0.686 to −0.733 in the outward open conformation and from −0.758 to −0.787 in the outward occluded conformation) were selected and for each of them the loop modeling protocol was applied. We used the loop modeling class available in MODELLER^43^, that samples various loop conformations on the defined region. Here, we selected a region of 5 residues, i.e. S477-T481, which contains the additional residue S479 and two amino acids framing the insertion. 100 loops were modeled for each of the 10 best models and scored with the Z-DOPE score. Thus 1000 models were generated for each conformation.

We then used the pi-helix detection program (i.e. pi_finder_WW.pl) on the 10 best-scored loops of each of the 10 models to filter the ones containing a π-helix around S479. This version of the program identifies pi-helices when two sequential i + 5 → I hydrogen bonds with energies < = −0.5 kcal/mol. We selected one final model for each conformation after visual inspection of each resulting model (Fig. 4).

### Model assessment

The final models in both conformations were evaluated with PROQM^44^ and PROCHECK^45^, two programs traditionally used to assess the quality of proteins structures. The outward open and outward occluded conformations present a PROQM score of 0.756 and 0.779 respectively, indicating good model quality. Furthermore, a PROCHECK analysis was run on the two models and showed that the backbones of the outward occluded and outward open conformation models displayed 93.1% and 94.3% of residues in acceptable regions of the Ramachandran plot. Overall, those criteria give us confidence in the global quality of our models to conduct structure-based studies.

### Induced fit docking and clustering

We conducted induced fit docking on our final model of CeaT derived from LeuT (PDB ID: 2A65). It is worth noting that the choice of the most appropriate model to conduct docking calculations is a balance between the quality of the model and the conformational state of the transporter. Here, despite the low sequence identity of the template with CreaT (i.e. 21%), the outward occluded conformation was chosen, as it is more likely to properly depict the interactions of the transporter with small molecules ligands. Furthermore, the PROQM and PROCHECK calculations show that both models (i.e. outward occluded and outward open) are of similar quality (see Model Assessment section).

The binding site grid was manually defined using Maestro^46^, selecting four amino acids predicted to be in the binding site of the creatine transporter: F68, C144, A318, G421. Mutations of these specific residues modulated the substrate specificity of the transporter. Specifically, a decrease of creatine uptake was concomitant to an increase of GABA uptake^24^. Altogether this suggests that these residues contribute in the binding site formation and their physico-chemical properties influence the ligand activities. Furthermore, the sequence alignment and fold prediction, together with the localization of the corresponding residues in the templates provide evidence of the participation of these residues to the binding site.

The protein was prepared within Maestro using Protein Preparation Wizard^47^ with default options. During this preparation the model was protonated. We then manually deprotonated C144 and the whole protein was energy-minimized. The five docked ligands (Table 1) were prepared with LigPrep^48^, with default options, except the pH range set at 7 ± 0.5.

Induced fit docking^49^ was performed using OPLS3e force field, no constrains were applied, a ligand conformational sampling within 2.5 kcal/mol, a van der Waals scaling of 0.5, 20 maximum number of poses, Prime refinement of residues within 5 Å of ligand poses, Glide redocking into structures within 30 kcal/mol of the best poses with a standard precision.

Finally, the poses presenting the best Emodel docking scores for each ligand were retained. In fact, Emodel combines several energy scoring functions (i.e. the GlideScore, the molecular mechanics interaction energy, and the internal ligand strain energy) and has been described as more appropriate than the GlideScore to compare conformers of the same molecule^50^. The GlideScore values are then used to compare poses of the distinct selected ligands.

### Binding affinities estimations

To rationalize the transport activities of CreaT ligands with their docking poses, we estimated the binding energies using Molecular Mechanics Generalized Born Surface Area (MMGBSA) solvation, implemented in the Schrödinger suite. Binding energies were calculated for the best pose (i.e. best Emodel score) of each CreaT-ligand complex resulting from the induced fit docking, and the defaults parameters implemented in the MMGBSA panel of the Schrödinger suite were used, including the VSGB 2.0^51^ energy model with the OPLS3e force field.
